# Morphology and mechanics of fungal mycelium

**DOI:** 10.1038/s41598-017-13295-2

**Published:** 2017-10-12

**Authors:** M. R. Islam, G. Tudryn, R. Bucinell, L. Schadler, R. C. Picu

**Affiliations:** 1 Department of Mechanical, Aerospace and Nuclear Engineering, Rensselaer Polytechnic Institute, Troy, NY 12180 USA; 2 Ecovative Design LLC, Green Island, NY 12183 USA; 3 Department of Mechanical Engineering, Union College, Schenectady, NY 12308 USA; 4 Department of Material Science and Engineering, Rensselaer Polytechnic Institute, Troy, NY 12180 USA

## Abstract

We study a unique biomaterial developed from fungal mycelium, the vegetative part and the root structure of fungi. Mycelium has a filamentous network structure with mechanics largely controlled by filament elasticity and branching, and network density. We report the morphological and mechanical characterization of mycelium through an integrated experimental and computational approach. The monotonic mechanical behavior of the mycelium is non-linear both in tension and compression. The material exhibits considerable strain hardening before rupture under tension, it mimics the open cell foam behavior under compression and exhibits hysteresis and the Mullins effect when subjected to cyclic loading. Based on our morphological characterization and experimental observations, we develop and validate a multiscale fiber network-based model for the mycelium which reproduces the tensile and compressive behavior of the material.

## Introduction

Engineering natural materials within the motif of sustainability and biodegradation has become a notable material design concept in recent years. Significant research effort has been devoted to developing materials from a wide range of natural resources such as cellulose^1^, silk protein^2,3^, eggshell membrane^4^, bamboo^5^, etc. Natural materials offer unique combination of properties that emerge predominantly from their intricate hierarchical and fibrous architecture^6,7^. While understanding the material chemistry is fundamental, delineating the mechanistic detail and the structure-property correlations underlying these properties is crucial for material design and optimization. Aligned with this motivation, we study here a unique biological material derived from fungal mycelium, the vegetative part and the root structure of fungi.

**Figure 1:**
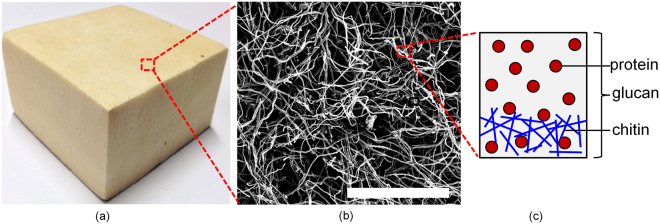
Mycelium at various length scales- (**a**) macroscale view (5 cm × 5 cm × 5 cm), (**b**) SEM image of the microstructure; scale bar 100 ${\rm{\mu }}{\rm{m}}$, and (**c**) schematic showing the structure of the single hyphae wall (Fig. 1(c) adapted from ref.^11^).

Mycelium has a porous structure composed of tubular filaments called hypha. Typically, hyphae have diameters on the order of 1–30 $\mu m$, depending on the species and growth environment, and lengths ranging from a few microns to several meters. Mycelium is one of the largest living organisms on Earth^8^. Mycelium grows out by apical tip expansion of hypha from a spore or an inoculum^9^. After an isotropic growth phase, hypha initiates random branching, forming fractal tree-like colonies^9^. Colonies interconnect randomly through hyphal fusion (anastomosis)^10^ to form a random fiber network structure (Fig. 1(b)). The branching density and network topology are largely controlled by the nutritional and environmental conditions.

The characteristic components of hypha wall are chitin, beta-glucans and proteins^11,12^ as shown in Fig. 1(c). The outer surface is rich in beta-glucans that serve as mucilage and the inner layer consists of chitin microfibils covalently cross-linked with other polysaccharides, such as glucans^11,12^. Chitin microfibrils provide the hypha its mechanical rigidity and strength. Stocks *et al*.^13^ and Zhao *et al*.^14^ reported elastic modulus of hyphae cell wall of two different species (*Saccharopolyspora erythraea* and *Aspergillus nidulans* respectively) in the range 110–140 MPa.

Microscopically, mycelium is a biopolymer network in which the mechanics is determined by individual filament behavior and the topological arrangement of filaments within the network. When subjected to far field loading, individual filaments rotate and deform in a manner dependent on their elastic properties, the orientation and connectivity within the network, leading to a complex global response of the network. Theoretical, experimental and computational studies on other network-based materials such as actin^15^ and collagen^16^ have demonstrated strong influence of network properties on macroscopic quantities^17,18^. Previous work on mycelium focused primarily on its chemistry which enabled its extensive used in biotechnological products^19^. Very few studies investigate the mechanical properties such as strength, stiffness and the structure-property relationships, which are relevant to a variety of applications of mycelium (e.g. as packaging, insulation or building material).

In this article, we report the morphological and mechanical characterization of mycelium through a synergetic combination of microscopic imaging, mechanical testing and computational modeling. Our experimental results reveal that mycelium exhibits significant non-linear stress-strain behavior both under tension and compression. We also observe that mycelium exhibits considerable strain hardening before rupture under tension and mimics open cell foam behavior under compression with strain dependent hysteresis and stress softening behavior under cyclic loading conditions. Based on these morphological characterization and experimental observations, we develop and validate a two-scale model of the material based on a random fiber network representation of the microstructure sequentially coupled with a stochastic continuum that captures material heterogeneity on scales larger than the microscale.

## Material and Methods

### Sample preparation

The mycelium samples were obtained from Ecovative Design, LLC. The general procedure used to grow the samples is as follows: the replicated mycelium vegetative tissue is introduced in a filter patch bag and allowed to grow by providing sufficient nutrition (calcium and carbohydrate) and water. This inoculation step takes 4 to 6 days. The inoculated substrate is then divided in small pieces to homogenize the mycelium growth and ensure uniform density throughout the material. After the homogenization is complete, the material is packed into tile molds and additional nutrition is provided to facilitate further growth. The tiles are allowed to grow for four additional days using proprietary environmental adjustments in temperature, humidity, oxygen and carbon dioxide to drive sufficient tissue growth from the surface of the samples. Sheets of fungal biomass are removed and dried at elevated temperature, for several hours, to deactivate the hyphae and stop the growth process. From these large samples, specimens of desired dimensions are cut for microscopic imaging and mechanical testing.

### Microscopic imaging

To evaluate the microstructural geometry of the mycelium, morphological characterization was carried out using a VERSA 3D Dual Beam Scanning Electron Microscope. SEM images were processed for calculating morphological statistics using image processing software, ImageJ^20^. Specifically, BoneJ^21^ plugin was used to estimate hypha diameter and pore size distribution.

### Mechanical testing

Uniaxial tension and compression tests were conducted using a MTS servo-hydraulic testing machine under displacement control and all the tests were performed in ambient conditions (25 °C and ~50% relative humidity). Dogbone specimens of dimensions 200 mm × 6 mm × 3.5 mm were used for tension tests, while compression tests were performed with cuboid specimens of dimensions 20 mm × 20 mm × 16 mm. All tensile tests were performed with a strain rate of 4 × 10^−4^ *s*^−1^ until failure, while the compressive specimens were deformed at a rate of 6.25 × 10^−3^ *s*^−1^ up to true strains ranging from 2% to 20%. Cyclic loading tests under both constant and increasing strain level in each cycle have been performed under compression. No hold/relaxation was applied between cycles.

### Digital image correlation (DIC)

A 3D DIC optical technique was used to monitor local strain field evolution in specimens during uniaxial compression testing. The specimen surfaces were sanded smooth and squared using 220 grit sand paper prior to testing. A stochastic speckle pattern, needed to perform the 3D-DIC, was applied to one surface of the specimen in a two-step process that insures proper speckle contrast and size. First, thinned flat white enamel paint (one part Model Master FS37875 Flat White to one part Model Master Air Brush Thinner) was lightly applied to the surface using a syphoning airbrush (Paasche VL0116). Second, a flat black paint (Model Master FS37038 without thinner) was applied to provide the contrasting speckles using a gravity-fed airbrush (Paasche Talon TG0516). Speckles size and density were adjusted using compressor pressure, distance from the airbrush to the specimen, needle position with respect to the venturi, and duration of speckling. The quality of the speckle pattern produced on each specimen was evaluated for correlation accuracy prior to the start of compression testing using the DIC software. Images were captured using two CCD cameras (2448 × 2050 pixel resolution) equipped with Schneider UNIFOC 2.8/50 lenses. Load and displacement data from the load frame were captured along with each image. ARMIS software by GOM was used to correlate images and calculate strains from displacement gradients.

### Data availability statement

The datasets generated during and/or analysed during the current study are available from the corresponding author on reasonable request.

## Experiments

### Morphology

**Figure 2:**
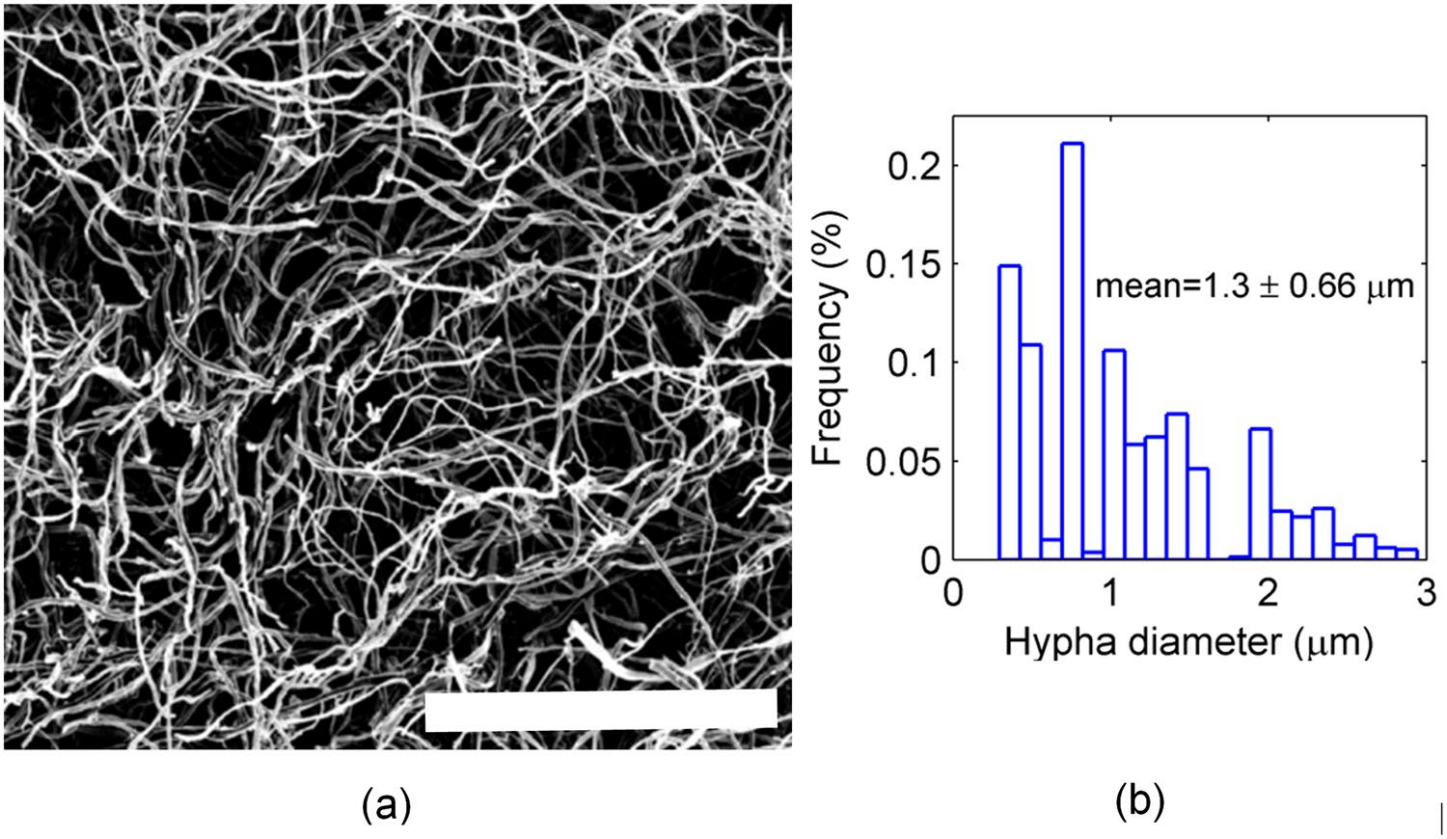
Morphological characterization of mycelium: (**a**) SEM image of mycelium (scale bar is 100 *μm*), (**b**) hyphae filament diameter distribution with mean 1.3 *μm*.

Figure 2(a) illustrates mycelium morphology observed in SEM. As noted above, the material exhibits a network like microstructure, with randomly arranged and oriented filaments. Image analysis was performed for multiple SEM images to estimate average network properties. The mean hypha filament diameter was 1.3 ± 0.66 *μm*. The corresponding distribution is shown in Fig. 2(b). The structure was statistically isotropic when inspected on three perpendicular planes.

### Uniaxial mechanical behavior in tension and compression

**Figure 3:**
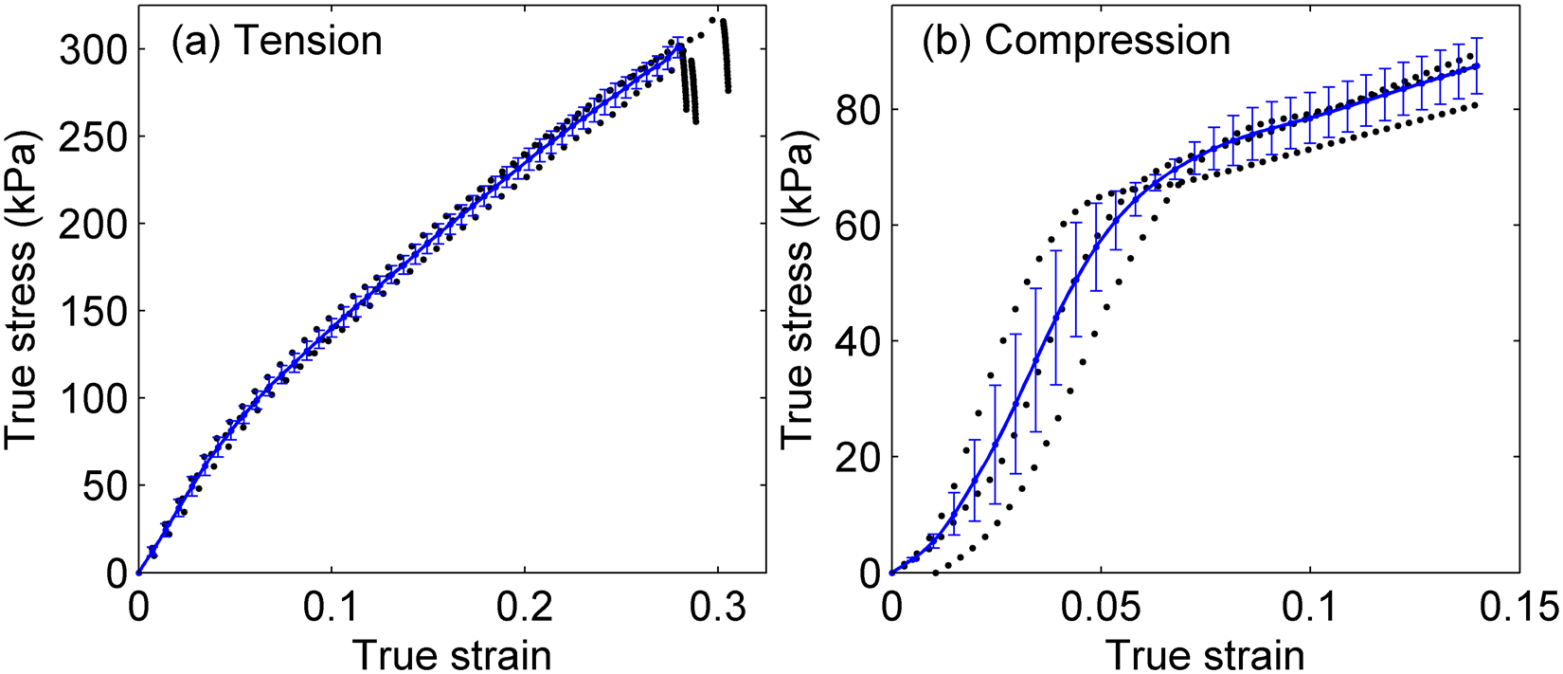
Generic stress-strain behavior of mycelium in (**a**) uniaxial tension and (**b**) compression (compressive stresses are shown as positive). The dotted lines (black) represent the range of tests performed on three specimens of comparable densities (〈*p*〉 ≈ 41 *kg*/*m*^3^) and the solid lines (blue) reperesent the corresponding mean responses (bars represent the standard deviation relative to the mean).

Figure 3(a) and (b) shows generic true stress-strain curves of mycelium under uniaxial tension and compression respectively. The dotted lines (black) represent the range of the stress-strain responses of three specimens of comparable density obtained from repeated tests, while the solid blue lines are the corresponding mean responses. The response is linear elastic at small strains, with the effective Young’s modulus being similar in tension and compression. At approximately 8% strain the material undergoes gradual softening in both cases, the softening being much more pronounced in compression. The tensile curve exhibits pronounced and almost linear strain hardening up to failure, which occurs abruptly at a strain of about 25%. The curve corresponding to compression develops a kink at about 8% strain followed by strain hardening which has a much smaller value than that measured in tension.

**Figure 4:**
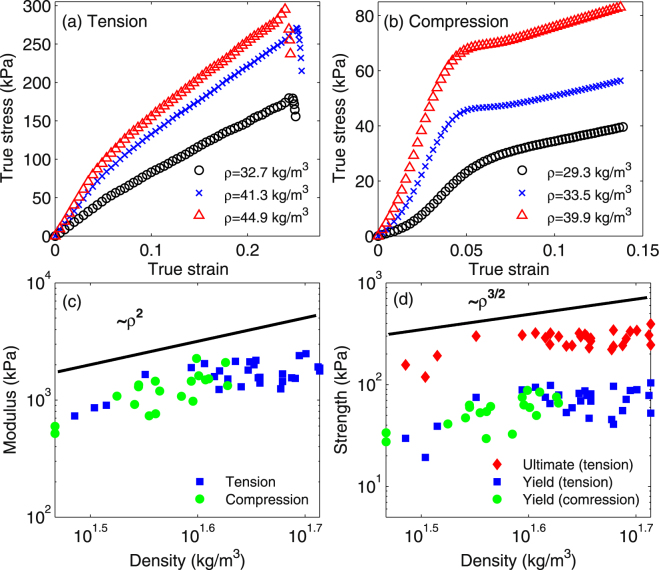
Effect of sample density on the stress-strain response in tension (**a**) and compression (**b**); Variation of the elastic modulus (**c**) and of the yield and ultimate tensile strength (**d**) with material density. Solid lines in (**c**,**d**) indicate the expected scaling for open cell foams.

The uniaxial stress-strain curves of the mycelium depend strongly on density. Figure 4(a) and (b) illustrate the stress-strain response of mycelium samples grown in slightly different conditions and having different densities. For cellular solids, both the modulus (${\rm{E}}$) and the ultimate tensile strength (${\rm{\sigma }}$) scale with the density ($\rho$) as: E ∝ E_s_(ρ/ρ_s_)^m^ and σ ∝ σ_s_(ρ/ρ_s_)^n^, respectively, where the subscript ‘s’ corresponds to bulk material properties of the cell walls and the exponents m and n depend on the cell geometry^22^. For open cell foams, the exponents are 2 for the elastic modulus and 1.5 for strength^22^. We measured the elastic modulus and yield strength of mycelium both in tension and compression by varying the sample density in the range from 30 kg/m^3^ to 50 kg/m^3^. We also measured the ultimate strength for samples tested in tension. Figure 4(c) and (d) illustrate the corresponding variation of the mycelium’s properties (modulus and strength) with the sample density for all samples. Modulus values obtained in tension and compression tests are reported. We observe that the modulus is approximately identical in tension and compression and varies from 600 to 2000 kPa for the given range of density. The yield stress (corresponding to the change of slope of the curves at about 8% strain) varies in the range of 40–80 kPa, whereas the ultimate tensile strength varies from 100 to 300 kPa.

**Figure 5:**
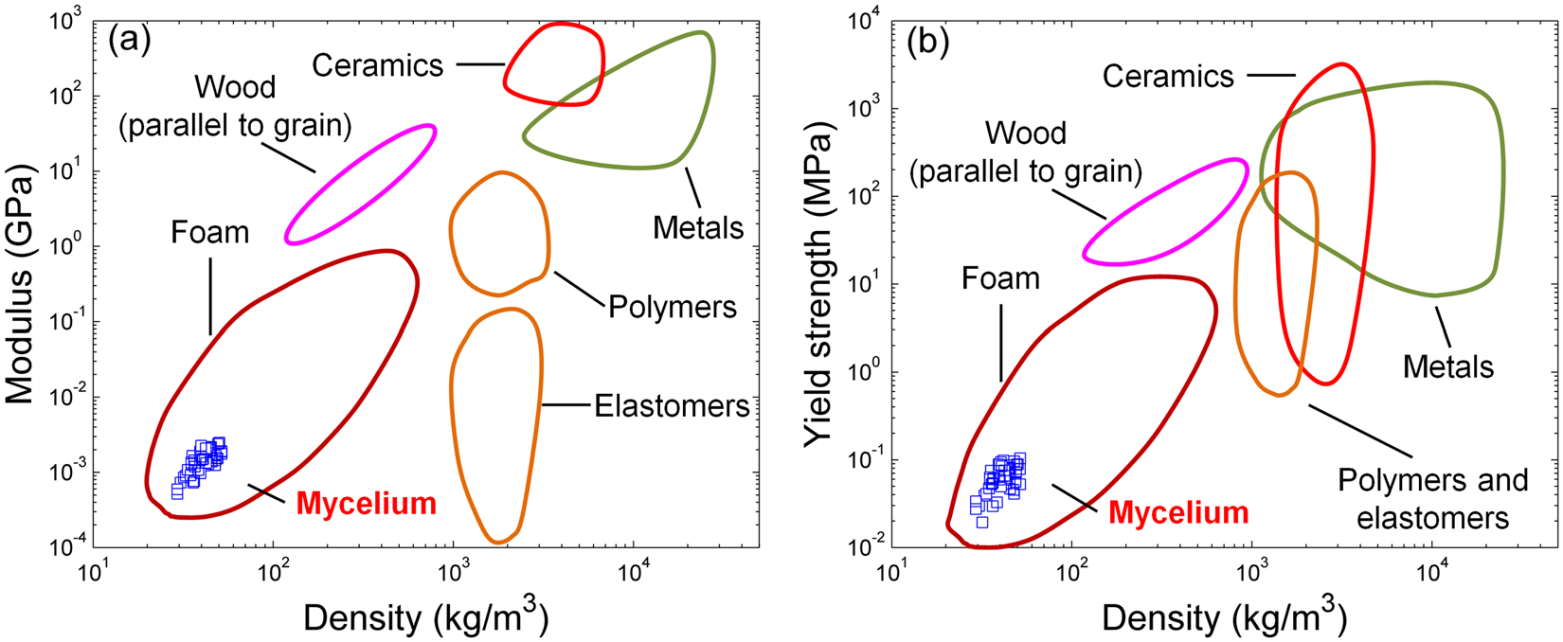
Comparison of mycelium mechanical properties with other traditional materials: (**a**) elastic modulus and (**b**) compressive yield strength versus density. Properties of materials other than the present mycelium results are illustrated by regions which are adapted from^27^.

Interestingly, the variation of all these quantities with the density is similar to that expected for open cell structures, despite clear differences between the network architecture shown in Fig. 2(a) and that of cellular materials (see ref.^22^ for other cellular structures). Precisely, we observe that the modulus varies with the square of mycelium density and strength varies with an exponent 3/2. Several researchers have reported similar scaling with density for other low density materials such as polymeric foams^23^, cancellous bone^24^ and collagen gels^25^. In Fig. 5, we compare mechanical properties of mycelium with other traditional materials using an Ashby map which demonstrates further that mycelium can be treated as biofoam material. The fields other than the present data are adapted from^26^.

### DIC strain measurement

As mycelium has a random filamentous microstructure, it is expected that its deformation is heterogeneous at the local scale. In addition, non-uniformities of the filament density distribution on the millimeter length scale and larger are introduced by the mycelium growth conditions. DIC measurements were performed in order to gain a quantitative understanding of the strain field heterogeneity induced by this structural heterogeneity.

**Figure 6:**
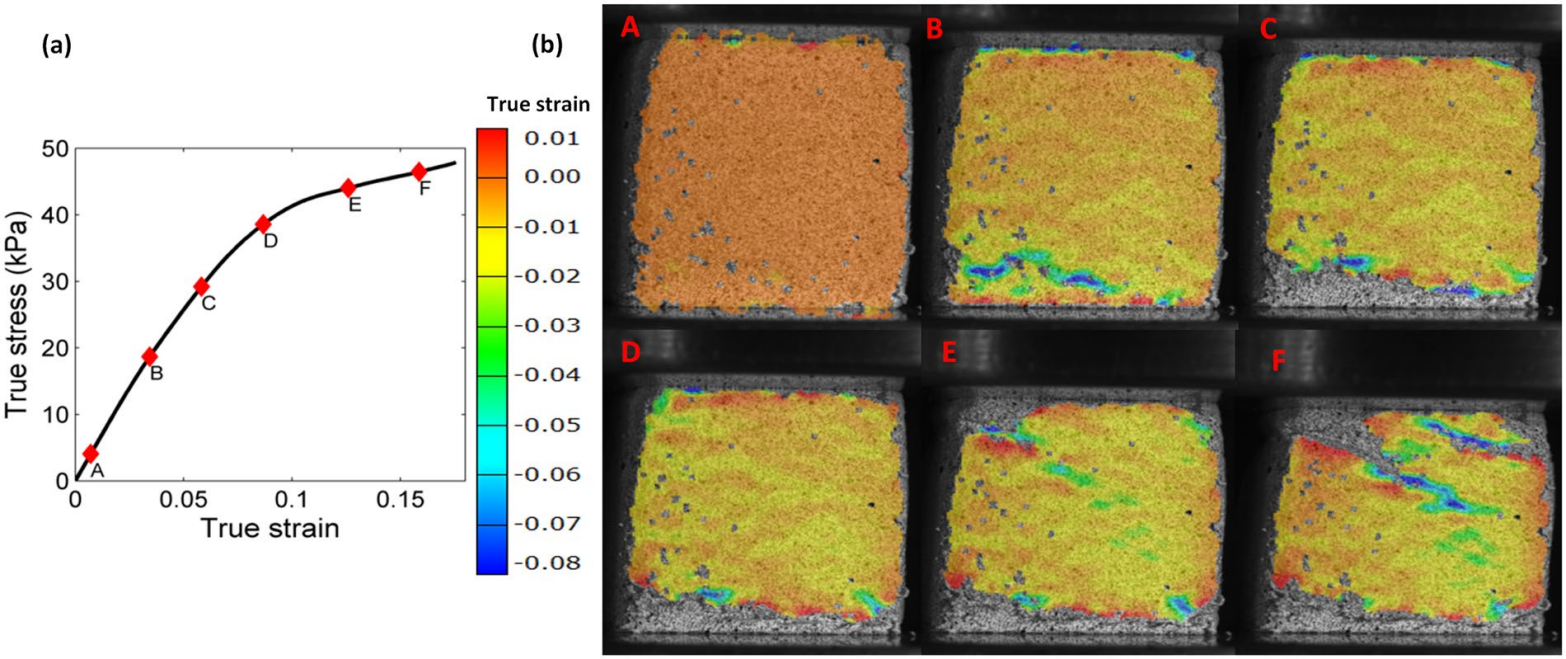
Strain evolution in mycelium during uniaxial compression: (**a**) global stress-strain curve, (**b**) sequence of DIC images corresponding to the points marked A to F on the stress-strain curve in (**a**). The color maps show the logarithmic normal strain in the loading (vertical) direction measured on the specimen surface.

Figure 6(a) shows the global macroscopic true stress-strain curve obtained during the compression experiment for which DIC images are selected. The red points A to F indicate the strains at which the images shown in Fig. 6(b) are taken. The maps represent the logarithmic normal strain component in the direction of the macroscopic load (vertical in these images). The local strain distribution is quite heterogeneous from the onset of deformation. At approximately 3% strain, localization initiates at the weakest sites in the specimen even though material is still in the linear regime at the macro scale. The strain localization becomes more pronounced as the macroscopic strain increases, percolating other weak domains in the vicinity and ultimately transforming into a collapse band (point D). The collapse band causes gradual degradation of the tangent modulus leading to a softer response in the global behavior. As the macroscopic strain increases, the formation of additional strain localization sites is observed (E) and these eventually merge into a band that traverses the specimen. The formation of multiple collapse bands associated with density fluctuations leads to the softening behavior observed beyond approximately 8% strain in compression. This is qualitatively similar to the response of cellular materials with non-periodic microstructures to compression.

### Cyclic compression behavior

**Figure 7:**
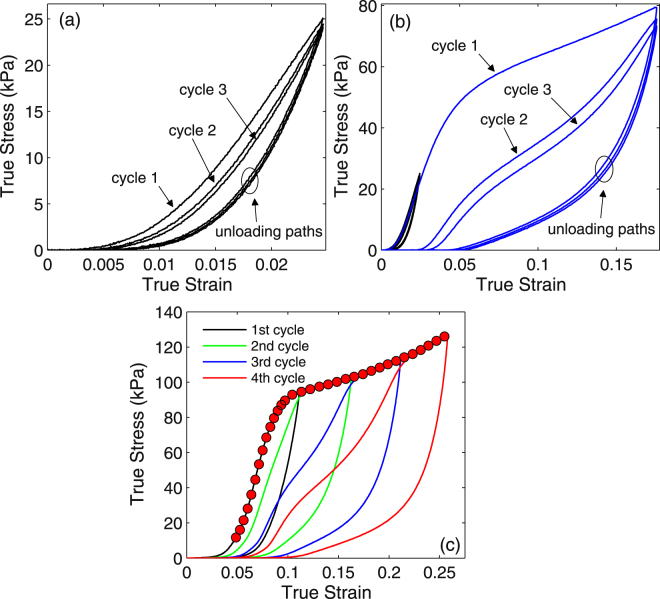
Compressive response of mycelium subjected to cyclic loading under constant strain amplitude ((**a,b**)) and increasing strain amplitude (**c**) at constant strain rate (6.25 × 10^−3^  s^−1^). The curve in (**a**) is reproduced in (**b**) for reference (black). The red dots in (**c**) represent the curve obtained for monotonic loading up to 25% strain.

Further, we investigate the cyclic compression response of mycelium under both constant and increasing maximum strain levels. Figure 7(a,b) show the true stress-strain response for three cycles of loading-unloading performed with imposed maximum strain of 2.2% (Fig. 7(a)) and 17% (Fig. 7(b)). Unloading is performed immediately after loading with the same strain rate (6.25 × 10^−3^ *s*^−1^). The curves in Fig. 7(a) are added to Fig. 7(b) for reference. The mycelium exhibits a pre-conditioning behavior similar to that reported for other materials with fibrous microstructure such as the dermis component of skin^28^. Specifically, the first cycle induces a substantial loss of stiffness, while the subsequent cycles have a smaller additional impact. Hysteresis is observed in all cases, probably due to the frictional interaction of filaments. The hysteresis is weak at small maximum strain amplitudes and increases progressively as the strain amplitude increases. If the strain amplitude is larger than 8% (beyond the yield point), the hysteresis is quite pronounced and a behavior similar to the Mullins effect^29^ is observed. The Mullins effect generally reported for unfilled or particle reinforced rubbers is associated with damage taking place in the material. In the case of the mycelium, strain localization occurs beyond the yield point. We conjecture that filament plasticity and non-affine kinematics of the filaments induced by these large local strains are responsible for the effect observed macroscopically. It is interesting that for cycles beyond the first cycle, the unloading paths approximately follow the same curve, independent of loading cycle number. Also, residual strain occurs predominantly after the first cycle.

Stress softening of mycelium was further explored by varying the strain amplitude in successive loading cycles. Figure 7(c) illustrates the true stress-strain responses from four loading and unloading cycles performed on the same specimen with strain amplitudes in subsequent cycles of ∈ = 0.11, 0.16, 0.21 and 0.26. No relaxation was allowed between the cycles. Cyclic stress softening and hysteresis are observed as reported above. Additionally, the upper envelope of the cyclic strain-stress curves coincides with the monotonic stress-strain curve obtained with a similar specimen (illustrated by red dots in Fig. 7(c). Progressive local network reorganization is observed in subsequent cycles, as the loading amplitude increases. This leads to large differences among the shapes of the reloading curves.

### Stress relaxation and strain rate effect

**Figure 8:**
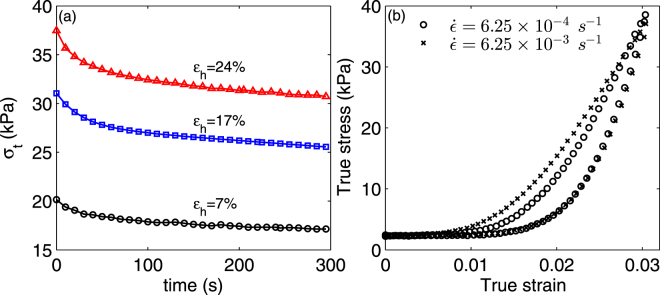
Stress relaxation behavior and rate sensitivity of mycelium: (**a**) stress vs. time curve for uniaxial compression at three different hold strain levels (7 % (black), 17 % (blue) and 24% (red)) and (**b**) uniaxial compressive response of mycelium at two different strain rates, $\mathop{{\rm{\epsilon }}}\limits^{\cdot }$ = 6.25 × 10^−3^ s^−1^ and $\mathop{{\rm{\epsilon }}}\limits^{\cdot }$ = 6.25 × 10^−4^ s^−1^).

To investigate the time dependence of the mechanical behavior, we performed stress relaxation tests under uniaxial compression. Figure 8(a) illustrates the evolution of the instantaneous stress (${\rm{\sigma }}({\rm{t}})$) as a function of time (t) at various holding strain levels (7%, 17% and 24% strain). Considerable stress-relaxation is observed. The stress drops rapidly in the initial part of the test followed by a gradual relaxation at longer time. Application of higher strain also leads to enhanced stress-relaxation as shown in Fig. 8(a). We also performed uniaxial compression test of mycelium at various strain rates. Figure 8(b) illustrates a compressive stress-strain curve of mycelium performed at strain rates of 6.25 × 10^−3^ s^−1^ and 6.25 × 10^−4^ s^−1^. To exclude the effect of material damage on the response, the tests were performed within small strains. The rate-dependence of the material behavior is not significant in this range of strain rates.

## Modeling mycelium mechanical behavior

In this section we present a two-scale model developed to represent the mechanical behavior of mycelium. The model can be further used to solve boundary value problems or to represent composites with mycelium matrix.

Given the network microstructure of the material, the most comprehensive model would be a random fiber network with fiber properties, structure and spatial distribution of fiber density matching those of the actual material. Such a model would include a very large number of fibers and would be intractable with the current modeling and simulation capabilities. To address this problem we develop a two-scale model. On the larger scale, comparable with the macroscopic scale of the samples tested, we use a stochastic continuum representation. The density and hence the mechanical behavior are allowed to change from sub-domain to sub-domain on this scale, with a characteristic length scale. This model takes into account density fluctuations with a length scale much larger than the network scale, i.e. the average distance between cross-links of the network. The constitutive behavior of each such sub-domain is provided by a sub-scale random fiber network model in which individual fibers are explicitly represented. This coupling is in the spirit of sequential multiscale models^30^. We present next each of these models, the coupling, the calibration, and the validation procedures.

### Network model

The microscale model is a representative volume element (RVE) of a random fiber network of specified density. The network is generated using a Voronoi tessellation algorithm. A set of randomly distributed seed points are generated inside a cubic domain and are used to generate a Voronoi tessellation. Fibers are defined along all edges of the resulting tessellation which results in an interconnected fiber network. The coordination number, i.e. the number of fiber segments emerging from each node, is z = 4. The networks density ($\rho )$ is defined as the total fiber length per unit volume and is equivalent to the mass density of the mycelium, ${\rho }_{m}$:

1 $${\rho }_{m}=(\frac{1}{V}){\rho }_{f}A\sum _{i=1}^{N}{l}_{i}=\rho {\rho }_{f}A$$

where V is the volume of the RVE, ${\rho }_{f}$ is the hyphae wall material density, A is the cross-section area of the tubular hyphae, N is the number of filaments in the network and ${l}_{i}$ is the length of the i^th^ filament.

**Figure 9:**
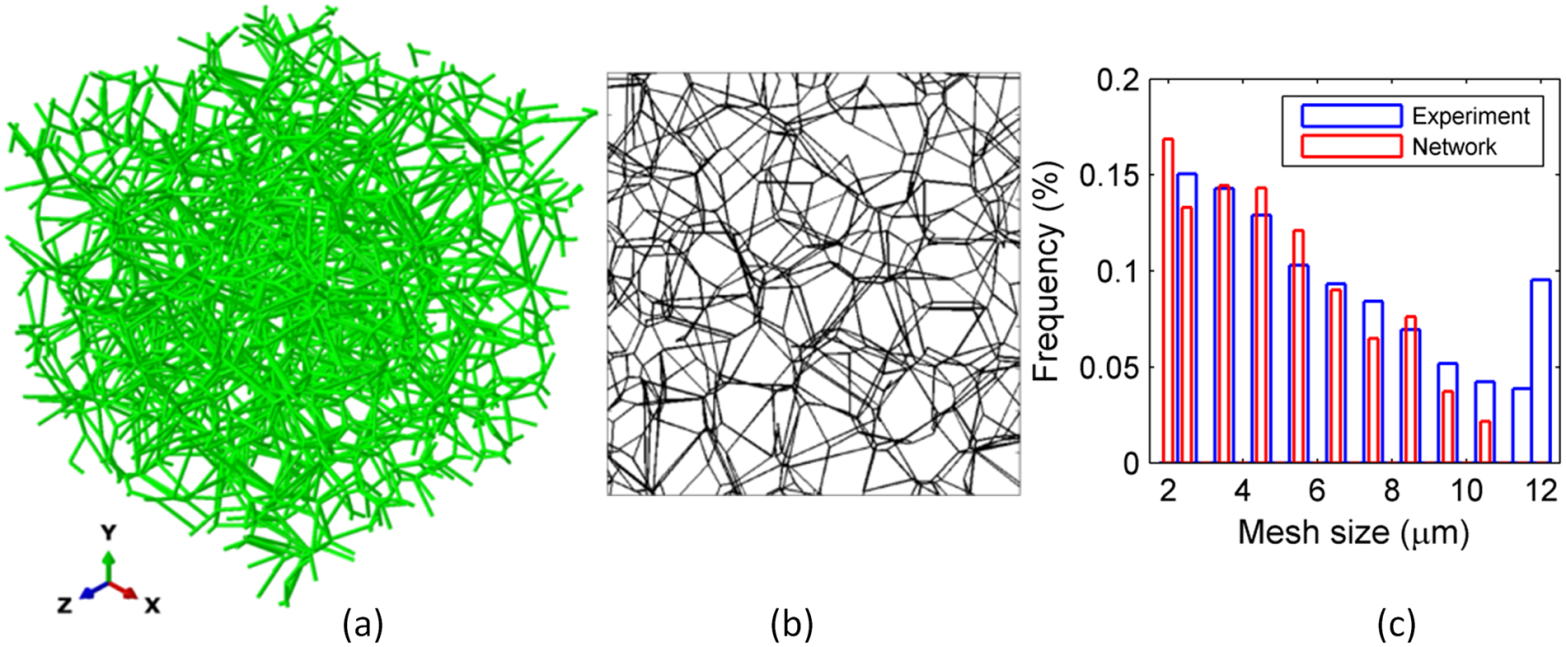
Random fiber network model: (**a**) representative network configuration, (**b**) projected view of a thin slice of the entire network and (**c**) comparison of the model network (red) and mycelium (blue) projected mesh size distributions.

The network density, $\rho$, is adjusted in the model by controlling the density of seeds used to generate the Voronoi tessellation of the domain. Therefore, two characteristic lengths are associated with the model: the size of the cubic RVE, L, and the average distance between two cross-links of the network, ${l}_{c}$. Parameter ${l}_{c}$ is inversely related to the network density, ${l}_{c} \sim 1/\rho$^31,32^. In order to eliminate size effects, L should be significantly larger than ${{l}}_{c}$. Specifically, we require that *L* ≥ 8*l*_*c*_, which is obtained by a separate analysis of size effects in these types of structures^33^. We used L = 100 $\mu m$ and ${l}_{c}$ varies in the range of 6–8 $\mu m$ for various densities. A representative network configuration generated using this procedure is illustrated in Fig. 9(a).

These relations allow calibration of the network density directly based on the mass density of the physical network. As an additional check of the resulting stochastic structure, we compare the topology of the model obtained using this procedure with the mycelium microstructure. To this end, we consider images of mycelium obtained by SEM (Fig. 2(a)). Given the finite depth of focus of the instrument, these images are actually 2D projections of the network structure within slices of the mycelium structure. These are compared with corresponding projections of slices of similar thickness of the model network. Figure 9(b) shows such a slice projection of the model network. The two projected structures are compared in terms of the mesh size distribution. Mesh size is calculated as the diameter of the greatest circle that fits within the void spaces in the 2D projected images. The corresponding mesh size distributions of the mycelium (mass density = 30 $\mathrm{kg}/{{\rm{m}}}^{3}$) and the corresponding model network are shown in Fig. 9(c). Each of these distributions is obtained by averaging over multiple replicas of the respective structure. The two distributions match closely, which indicates that the geometry of the model network reproduces the measurable features of the actual mycelium network and no calibration is needed. The mean of the two distributions is 5 $\mu m$.

In the actual mycelium, the diameter of individual filaments varies in the range from 0.2 ${\rm{\mu }}{\rm{m}}$ to 3 ${\rm{\mu }}{\rm{m}}$ (Fig. 2b). However, all filaments in the network model are assumed to have identical cross-section with outer diameter d_o_ = 1.3 *μm* (Fig. 2 (b)) and wall thickness (t_w_ = 100 *nm*)^14^. The base material of the filament is chitin which has density ρ_f_ = 1430 kg/m^3^. The mechanical behavior of the chitin wall is considered elastic-plastic with the elastic modulus, Poisson ratio and yield strength reported in the literature for chitin, 2.5 GPa, 0.3 and 45 MPa respectively^34^. In the finite element model constructed based on the structure of the RVE (Fig. 9(a)) filaments are represented using Timoshenko beams which store strain energy in the axial, bending and shear modes. The number of elements per fiber is selected such to keep the element aspect ratio close to 5. The model is subjected to boundary conditions that mimic the experimental set-ups and solved using the general purpose finite element solver Abaqus (version 6.13-1).

In uniaxial tension, top boundary nodes are subjected to prescribed displacement in the loading direction while bottom boundary nodes are constrained in the loading direction. To study the network behavior in compression, we introduce two rigid surfaces at the top and bottom boundaries of the network and network is compressed by displacing the top surface while keeping the bottom surface stationary. Additionally, in compression, we incorporate surface based contact between the rigid surfaces and beam element surfaces similar to^35^.

**Figure 10:**
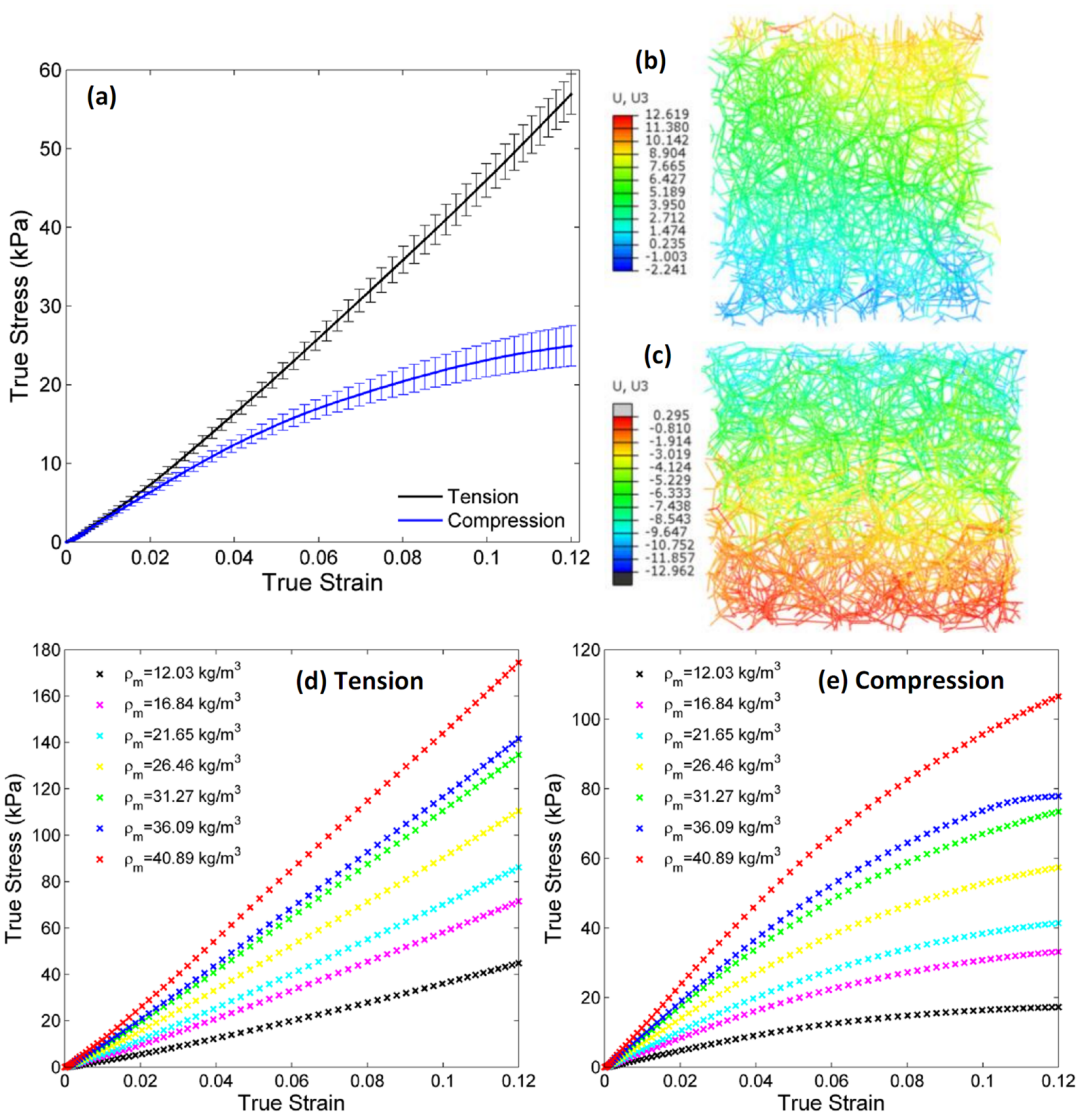
3D random fiber network behavior: (**a**) representative stress-strain response (bars represent the range of three realizations) of a network with mass density ρ_*m*_ = 16.84 kg/m^3^ in tension (black) and compression (red) with bars indicating the range of three realizations; Deformed network configurations with displacement contours in (**b**) tension and (**c**) compression; Stress-strain curves (averaged over three realizations) for seven different densities for (**d**) tensile and (**e**) compressive loading.

Figure 10(a) illustrates a representative true stress-true strain response in tension and compression (the bars represent the range of three realizations) for a network RVE of density ρ_*m*_ = 16.84 kg/m^3^. At small strains the network exhibits a linear elastic response and the stiffness is identical in tension and compression. This is in agreement with experimental observations (Fig. 4(c)). The curve corresponding to compression softens starting from approximately 5% strain. This is due to the pronounced bending of filaments and their reorientation in the direction perpendicular to the compressive axis. The curve corresponding to tension stiffens slightly once the strain is larger than 5% due to preferential fiber orientation in the loading direction. Representative deformed network configurations with displacement contours are illustrated in Fig. 10(b) and (c) under tension and compression respectively, at 10% global strain demonstrating strain non-uniformity in this stochastic structure. Figure 10(d,e) illustrates the stress-strain response of networks with seven different densities (averaged over three realizations) under tension and compression respectively (each curve is an average over 3 realizations). The curves show qualitatively similar behavior as discussed above.

### Continuum model

**Figure 11:**
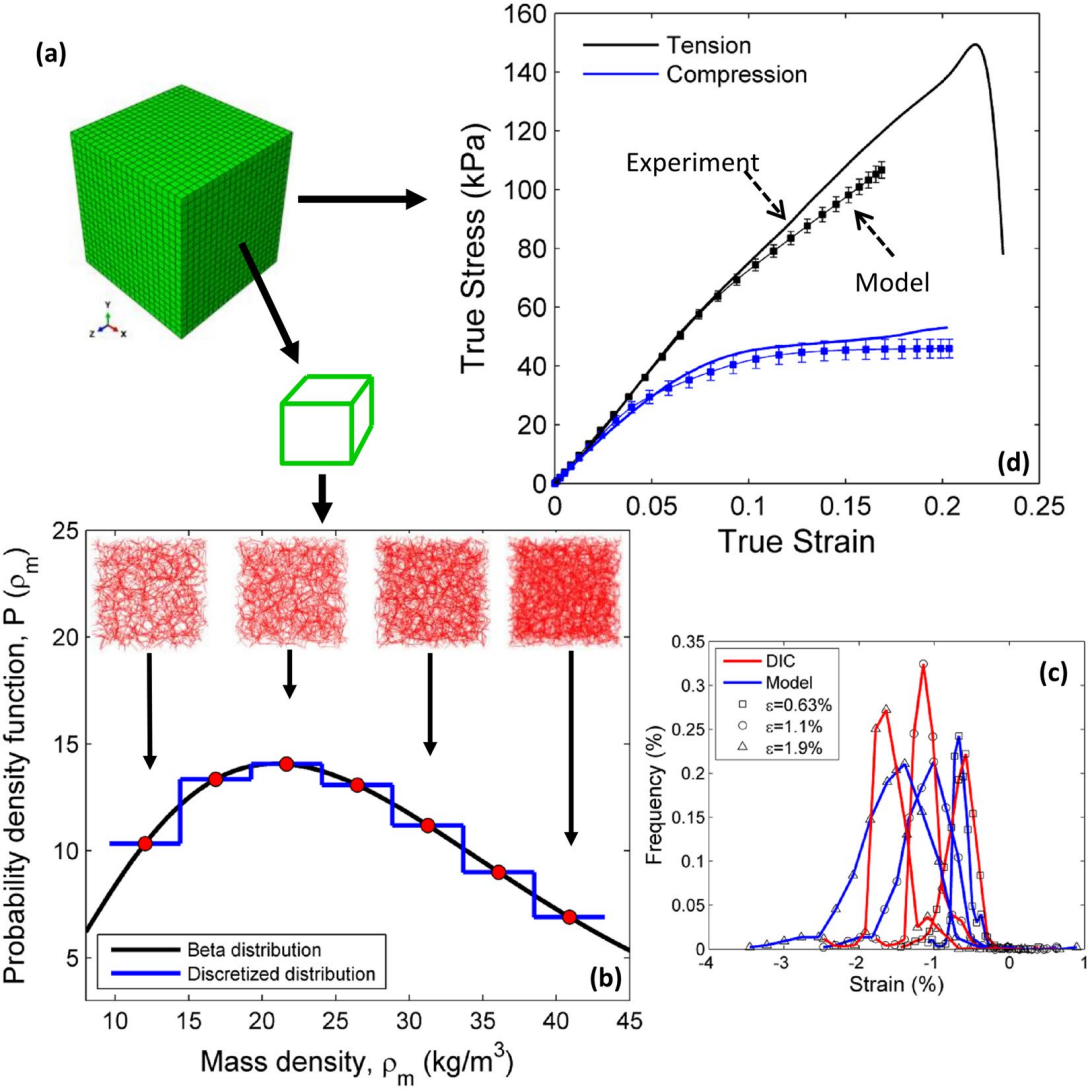
Procedure used to map the microscale network behavior to subdomains of the continuum stochastic macroscale model. (**a**) Finite element model containing 8000 subdomains; (**b**) Probability distribution function of mass density resulting from by fitting the DIC strain distribution and its discretization in 7 distinct ranges of density. The insets show realizations of the network microscale RVEs corresponding to each of the 7 density bins; (**c**) Comparison of normal strain distribution measured on the surface of the physical sample (red) and macroscopic model (blue) corresponding to the mass density distribution in (**b**) for three values of the far field applied strain in the linear response regime of the mycelium; (**d**) Comparison of global stress-strain response predicted by the model (symbols) and experiments (solid lines); Error bars indicate range of five realizations.

A 3D stochastic continuum model is used at the macroscale to obtain a homogenized representation of the macroscopic mycelium mechanical behavior, Fig. 11(a). The domain is divided in 8000 subdomains, each being assigned a network density sampled from a distribution. In principle, the constitutive behavior of each such sub-domain would be provided by a microscale RVE loaded with the strain of the respective macroscale sub-volume. This procedure is computationally expensive and requires tracing the deformation history of each element. To render the method more computationally efficient while being able to effectively account for microstructural variability, we pre-average the response to tension/compression of separate microscale RVEs. RVEs of seven different densities are considered (Fig. 10(d) to (e)). These numerically defined constitutive laws are then assigned randomly to sub-domains of the continuum model. The volume fractions corresponding to each density correspond to the distribution of densities of the actual mycelium.

In order to identify the fluctuations of density on the mesoscale, i.e. at a scale much larger than the mesh size of the network, but smaller than the experimental sample size, we keep the density distribution (specifically, the fractions representing the seven densities selected) as a parameter, construct macroscale models for each realization of this ensemble, load the resulting model in compression and compare the resulting strains with the strain distribution obtained from DIC. We iterate through this procedure until the distribution of computed strains matches the distribution of measured strains. Figure 11 summarizes the procedure and the results. We consider that the distribution of mass density in the mycelium follows a beta distribution, Fig. 11(b). The mass density values are assigned to the sub-domains of the continuum model in an uncorrelated way. This is identical to requiring that the correlation length of the mass density distribution in the physical sample is equal to the size of the subdomains considered. The mean of the distribution is equal to the actual macroscopic sample density. This leaves only the variance of the distribution to be identified based on the procedure stated above. Figure 11(c) shows the probability distribution functions for the normal strains measured on the surface of the mycelium sample in the loading direction with DIC, and computed with the continuum model at the end of this optimization procedure. Distributions are shown for three levels of macroscopic strain in the linear elastic range. This corresponds to the density distribution function shown in Fig. 11(b) which has a coefficient of variation of 0.48 ${kg}/{m}^{3}$ and which, according to the procedure used here, is considered to match the density distribution in the tested physical sample with macroscopic density (*ρ* = 30 kg/m^3^).

Once the density distribution is defined, the subdomains are being assigned one of the constitutive behaviors shown in Fig. 10(d,e). Specifically, we used an elastic-plastic material model with parameters such as elastic modulus, yield strength calculated from the network stress-strain responses as shown in Fig. 10(d) and (e).

Figure 11(d) shows the comparison of the measured and predicted macroscopic true stress- true strain behavior in both tension and compression. The curves represent the average of five realizations of the continuum macroscale model and the bars represent the range of the respective replicas. The model prediction is identical to that of mycelium in the respective strain range. It is possible that in the physical sample damage of hyphae takes place during deformation. In absence of reliable damage properties available for hyphae filaments, we choose not to incorporate damage into the network model. In compression, the model predicts accurately the onset of localization and the strain hardening beyond the yield point. It is emphasized that this is a prediction of the model and was not fitted. The only calibration needed was that of the distribution of densities in the continuum model.

## Conclusion

We presented morphological and mechanical characterization of a novel biomaterial derived from fungal mycelium. The experimental results revealed the most significant characteristics of mycelium under tension and compression. In tension, the material response is linear elastic at low strain, and then the material yields and undergoes strain hardening before rupture. On the other hand, the bio polymer behaves similar to open cell foam under uniaxial compression, where the stress-strain curve shows an initial linear-elastic regime followed by a plateau regime with softened response. Furthermore, when subjected to successive loading and unloading cycles, mycelium exhibits strain dependent hysteresis and stress softening effect (Mullins effect) from cycle to cycle. The mechanical properties show significant variation with material density. The elastic modulus results in the range 600 to 2000 kPa for the given range of densities for both in tension and compression. The measured yield strength is in the order of 40–80 kPa, whereas the ultimate strength in tension varies from 100–300 kPa depending on material density.

A multiscale continuum model incorporating microstructural details of the network and which accounts for spatial density variation in the actual material was developed. It is demonstrated that the model represents the topology of mycelium at the network scale. By comparing the strain distribution in the model with the strain distribution measured through DIC, we calibrate the distribution of density in the material on the mesoscale. The stress-strain curves on the mesoscale are uniquely defined by the local density and fiber properties obtained from the literature. The stochastic continuum model taking into account density fluctuations predicts global stress-strain curves in close agreement with the experimental results in both tension and compression. This model can be used to investigate the mechanical behavior of mycelium based composites, which represents the next stage of this investigation.
