# Publisher Correction: Morphology and mechanics of fungal mycelium

**DOI:** 10.1038/s41598-018-20637-1

**Published:** 2018-03-06

**Authors:** M. R. Islam, G. Tudryn, R. Bucinell, L. Schadler, R. C. Picu

**Affiliations:** 1 Department of Mechanical, Aerospace and Nuclear Engineering, Rensselaer Polytechnic Institute, Troy, NY 12180 USA; 2 Ecovative Design LLC, Green Island, NY 12183 USA; 3 Department of Mechanical Engineering, Union College, Schenectady, NY 12308 USA; 4 Department of Material Science and Engineering, Rensselaer Polytechnic Institute, Troy, NY 12180 USA

Correction to: *Scientific Reports* 10.1038/s41598-017-13295-2, published online 12 October 2017

The original PDF version of this Article contained errors. Figures 3, 4 and 7 were duplicated in Figures 4, 6 and 10 respectively, Figures 4–8 were incorrectly listed as Figures 5, 7, 8, 9 and 11 respectively and Figures 9–11 were missing.

These errors have now been corrected in the PDF version of this Article; the HTML version was correct from the time of publication.

